# Supplementary material for: An international comparison of factors affecting quality of life among patients with congestive heart failure: A cross-sectional study
Source: PLoS One. 2020 Apr 8;15(4):e0231346. doi: 10.1371/journal.pone.0231346 (PMC7141662; doi:10.1371/journal.pone.0231346)
Supplement: S1 File — (PDF) [file pone.0231346.s001.pdf]

ID

Draft

IQ Scientific Center for Quality of Healthcare

RHeLaunCh

Reframing Healthcare through the Lens of Coproduction

Vragenlijst

Over de gezondheid, leefsituatie, zorggebruik, -behoeften en -ervaringen van patiënten met COPD en chronisch hartfalen in Nederland en de Verenigde Staten

IQ healthcare, maart/april 2017

Draft

ID

Instructies bij de vragenlijst

- Kies per vraag steeds het antwoord dat volgens u het juiste is of het beste bij uw situatie past.
- Lees de vraag eerst goed door vóór u uw antwoord invult.
- Wilt u de vragen met een donkere pen (blauw of zwart) invullen?
- Zet de kruisjes alstublieft in de vakjes. De vragenlijst wordt namelijk machinaal ingelezen en verwerkt. Informatie die naast de vakjes staat wordt niet verwerkt.
- Als u een antwoord wilt verbeteren, maak dan het 'foute' vakje helemaal donker en kruis het nieuwe antwoord aan, zoals in het voorbeeld hieronder. Maak alstublieft geen gebruik van Tipp-ex.

Voorbeeld: verbeteren van een antwoord

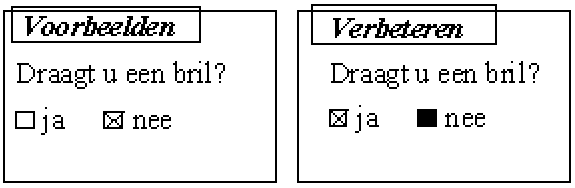

Voor dit onderzoek is het erg belangrijk dat u bij **alle** vragen een antwoord invult door één antwoord aan te kruisen.

Na het invullen
Wanneer u de vragenlijst heeft ingevuld, kunt u de ingevulde lijst terugsturen naar het Radboudumc met de bijgevoegde antwoordenveloppe. Een postzegel is niet nodig.

Vragen?
Indien u vragen of opmerkingen heeft, dan kunt u contact opnemen met:

Dr. Gijs Hesselink (tel. 024-3666264/ Gijs.Hesselink@radboudumc.nl)
Drs. Wytske Geense (tel. 024-3666868/ Wytske.Geense@radboudumc.nl)
Mw. Lisette Baltussen (tel. 024-3692767/ Lisette.Baltussen@radboudumc.nl)

Postadres:
Radboudumc
114 IQ Healthcare
Antwoordnummer 540
6500 VC Nijmegen

Alvast hartelijk dank voor het invullen!

Draft

ID

A. Algemene gegevens

1. Wat is uw geboortejaar? [ ][ ][ ][ ]

2. Bent u een man of vrouw? ☐ Vrouw ☐ Man

3. Wat is uw gewicht? [ ][ ][ ] kg

4. Wat is uw lichaamslengte? [ ][ ][ ] cm

5. Bent u naast COPD of hartfalen voor andere ziekten/aandoeningen bij een arts onder behandeling?
   - ☐ Nee
   - ☐ Ja, namelijk: ....

6. Gebruikt u zes of meer verschillende soorten voorgeschreven medicijnen?
   - ☐ Nee
   - ☐ Ja
   - ☐ Weet ik niet

7. In welk land bent u geboren, en in welk land zijn uw vader en moeder geboren?

Geboorteland van:	Uzelf	Uw vader	Uw moeder
Nederland	☐	☐	☐
Turkije	☐	☐	☐
Indonesië/voormalig Nederlands-Indië	☐	☐	☐
Suriname	☐	☐	☐
Marokko	☐	☐	☐
Nederlandse Antillen en Aruba	☐	☐	☐
Anders:	☐	☐	☐

8. Wat is uw huidige burgerlijke staat?
   - ☐ Alleenstaand/ongehuwd
   - ☐ Gehuwd
   - ☐ Geregistreerd partnerschap/samenlevingscontract
   - ☐ Gescheiden
   - ☐ Weduwe/weduwnaar

9. Vindt u het goed dat we uw postcodecijfers gebruiken voor ons onderzoek? We kunnen hiermee bijvoorbeeld vaststellen of u in een landelijk of stedelijk gebied woont en of u dichtbij of veraf woont van het ziekenhuis en de apotheek. Wij geven uw postcodecijfers niet door aan derden.

   Mijn postcodecijfers zijn: [ ][ ][ ][ ]

10. Heeft u een **basis** zorgverzekering?

☐ Nee

☐ Ja

☐ Weet ik niet

11. Heeft u een **aanvullende** zorgverzekering?

☐ Nee

☐ Ja

☐ Weet ik niet

12. Mijn huidige woning/verblijf:

☐ …is door mij of iemand anders in mijn huishouden gekocht

☐ …wordt door mij of iemand anders in mijn huishouden gehuurd

☐ …wordt door mij bewoond zonder huur- of hypotheekkosten

☐ Ik heb geen vaste verblijfplaats/ ik ben dakloos

☐ Anders, namelijk: ....

13. Kunt u aangeven welke mensen op dit moment deel uitmaken van uw huishouden?
(meer dan één antwoord mogelijk)

☐ Geen andere personen buiten mijzelf

☐ Partner/ echtgeno(o)t(e)

☐ Kinderen (inwonend)

☐ Ouder(s)

☐ Kind bij wie ik inwoon

☐ Anders, namelijk: ....

14. Wat is de hoogste opleiding die u heeft afgerond?
(één antwoord aankruisen)

☐ Geen (lagere school niet afgemaakt)

☐ Lagere school/ basisschool

☐ Huishoudschool, LTS, LEAO, LHNO, VMBO

☐ (M)ULO, MAVO, MBO, drie jaar HBS

☐ HAVO, HBS, Atheneum, Gymnasium

☐ HBO, HTS, HEAO

☐ Universiteit Bachelor

☐ Universiteit Master

☐ Doctoraat

☐ Anders, namelijk: ....

15. Welke omschrijving is op dit moment **het meest** op u van toepassing?
(één antwoord aankruisen)

- ☐ Voltijd (fulltime) baan
- ☐ Deeltijd (parttime) baan
- ☐ Volledig of gedeeltelijk afgekeurd
- ☐ Werkloos
- ☐ Huisvrouw/-man
- ☐ Gepensioneerd
- ☐ Vrijwilliger
- ☐ Anders, namelijk: ....

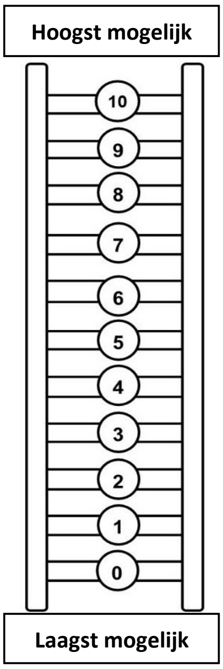

16. Hiernaast ziet u een ladder. De ladder geeft weer hoe mensen tegen hun leven aankijken: de hoogste trede (10) staat voor het best mogelijke leven.
De laagste trede (0) staat voor het slechtst mogelijke leven.
Op welke trede van deze ladder ziet u uzelf staan?

0	1	2	3	4	5	6	7	8	9	10
☐	☐	☐	☐	☐	☐	☐	☐	☐	☐	☐

17. Kijkt u alstublieft nog een keer naar de ladder. De ladder geeft nu uw financiële situatie weer: de hoogste trede (10) staat voor de best mogelijke financiële situatie.
De laagste trede (0) staat voor de slechtst mogelijke financiële situatie.
Op welke trede van deze ladder ziet u uzelf staan?

0	1	2	3	4	5	6	7	8	9	10
☐	☐	☐	☐	☐	☐	☐	☐	☐	☐	☐

B. Lichamelijke gezondheid, leefstijl en dagelijks functioneren

18. Hoe beoordeelt u uw lichamelijke gezondheid over het geheel genomen?

- ☐ Uitstekend
- ☐ Goed
- ☐ Voldoende
- ☐ Matig
- ☐ Slecht

18a. Voor onderstaande vraag is het belangrijk om te weten of u hartfalen of COPD heeft.

☐ Ik heb hartfalen (ga naar vraag 18b)

☐ Ik heb COPD (ga naar vraag 18c)

18b. Welke van de onderstaande uitspraken is voor u het meest van toepassing?
(één antwoord aankruisen)

☐ Geen last van kortademigheid of vermoeidheid.

☐ Kortademigheid of vermoeidheid bij normale lichamelijke activiteit.

☐ Kortademigheid of vermoeidheid bij geringe inspanning.

☐ Kortademigheid of vermoeidheid bij elke inspanning en in rust.

18c. Welke van de onderstaande uitspraken is voor u het meest van toepassing?
(één antwoord aankruisen)

☐ Ik word alleen kortademig bij zware inspanning.

☐ Ik word alleen kortademig als ik me moet haasten op vlak terrein of tegen een lichte helling op loop.

☐ Door mijn kortademigheid loop ik op vlak terrein langzamer dan andere mensen van mijn leeftijd, of moet ik stoppen om op adem te komen als ik mijn eigen tempo loop.

☐ Na ongeveer 100 meter lopen op vlak terrein moet ik na een paar minuten stoppen om op adem te komen.

☐ Ik ben te kortademig om het huis uit te gaan, of ik ben kortademig tijdens het aan- of uitkleden.

19. Hoe vaak heeft u in het afgelopen jaar gerookt (sigaretten, elektrische sigaretten, sigaren, pijp, shag, pruimtabak, marihuana, etc.)?

☐ Dagelijks of bijna dagelijks

☐ Wekelijks

☐ Maandelijks

☐ Minder dan één keer per maand

☐ Nooit

20. Hoe vaak heeft u in het afgelopen jaar **5 of meer glazen alcoholhoudende drank per dag** gedronken?

☐ Dagelijks of bijna dagelijks

☐ Wekelijks

☐ Maandelijks

☐ Minder dan één keer per maand

☐ Nooit

21. Hoe vaak heeft u in het afgelopen jaar soft- en/of harddrugs (zoals marihuana, cocaïne, heroïne, MDMA, XTC, hallucinerende middelen, etc.) gebruikt?

☐ Dagelijks of bijna dagelijks
☐ Wekelijks
☐ Maandelijks
☐ Minder dan één keer per maand
☐ Nooit

22. Hoe vaak heeft u in het afgelopen jaar medicijnen gebruikt om uw stemming te beïnvloeden (zoals antidepressiva, slaappillen, sterke pijnstillers, etc.), terwijl deze medicijnen niet aan u waren voorgeschreven?

☐ Dagelijks of bijna dagelijks
☐ Wekelijks
☐ Maandelijks
☐ Minder dan één keer per maand
☐ Nooit

23. Kunt u aangeven hoeveel last u de afgelopen vier weken heeft gehad met het uitvoeren van deze dagelijkse activiteiten?

	**Geheel geen last**	**Erg weinig last**	**Een beetje last**	**Redelijk wat last**	**Flink wat last**	**Erg veel last**	**Heel erg veel last**
a) Traplopen	☐	☐	☐	☐	☐	☐	☐
b) Een stukje hardlopen	☐	☐	☐	☐	☐	☐	☐
c) Fietsen	☐	☐	☐	☐	☐	☐	☐
d) Iets zwaars optillen	☐	☐	☐	☐	☐	☐	☐
e) Naar het toilet gaan	☐	☐	☐	☐	☐	☐	☐
f) Baden/douchen en/of aankleden	☐	☐	☐	☐	☐	☐	☐
g) Schoonmaken	☐	☐	☐	☐	☐	☐	☐
h) Voor het gezin zorgen	☐	☐	☐	☐	☐	☐	☐
i) Winkelen/boodschappen doen	☐	☐	☐	☐	☐	☐	☐

24. Als u denkt aan het behouden en/of het verbeteren van uw **lichamelijke gezondheid,** zoals fitheid, bewegen, klachten zoals pijn en kortademigheid:

a) Krijgt u hierbij hulp?
☐ Ja *(ga naar vraag b)*
☐ Nee *(ga naar vraag c)*

b) Sluit de hulp die u krijgt goed aan bij wat u nodig heeft?
☐ Ja
☐ Nee

c) Wilt u hierbij hulp?
☐ Ja
☐ Nee

Draft

C. Psychische gezondheid en welbevinden

25. Als u denkt aan het behouden en/of het verbeteren van uw **dagelijks functioneren**, zoals persoonlijke verzorging, werken, volgen van een opleiding, winkelen, hobby's, huishouden:

a) Krijgt u hierbij hulp?

- ☐ Ja *(ga naar vraag b)*
- ☐ Nee *(ga naar vraag c)*

b) Sluit de hulp die u krijgt goed aan bij wat u nodig heeft?

- ☐ Ja
- ☐ Nee

c) Wilt u hierbij hulp?

- ☐ Ja
- ☐ Nee

26. Hoe beoordeelt u uw psychische gezondheid over het geheel genomen, inclusief uw humeur en denkend vermogen?

- ☐ Uitstekend
- ☐ Goed
- ☐ Voldoende
- ☐ Matig
- ☐ Slecht

27. De wijze waarop mensen in het leven staan en hoe zij met stress en tegenslagen omgaan kan van invloed zijn op hun gezondheid en kwaliteit van leven.

Kunt u aangeven in welke mate de volgende uitspraken passen bij uw houding en gedrag?

	Sterk mee eens	Mee eens	Neutraal	Mee oneens	Sterk mee oneens
a) Ik heb het gevoel dat ik iets kan betekenen voor iemand anders.	☐	☐	☐	☐	☐
b) Ik heb een doel in mijn leven.	☐	☐	☐	☐	☐
c) Ik haal voldoening uit wat goed gaat.	☐	☐	☐	☐	☐
d) Ik weet wel raad met de problemen die op mijn weg komen.	☐	☐	☐	☐	☐
e) Ik beslis hoe ik baas word over mijn leven.	☐	☐	☐	☐	☐
f) Ik heb de wil om verder te gaan.	☐	☐	☐	☐	☐
g) Mijn chronische ziekte bepaalt niet wie ik ben.	☐	☐	☐	☐	☐
h) Ik vind mezelf de moeite waard.	☐	☐	☐	☐	☐
i) Ik zet negatieve gedachten om in positieve gedachten.	☐	☐	☐	☐	☐
j) Ik overzie hoe mijn leven mij gevormd heeft tot wie ik nu ben.	☐	☐	☐	☐	☐
k) Ik kan omgaan met mijn kwetsbaarheden.	☐	☐	☐	☐	☐
l) Ik durf op mezelf te vertrouwen.	☐	☐	☐	☐	☐

Draft

ID

28. Als u denkt aan het behouden en/of het verbeteren van **uw psychische gezondheid,** zoals vrolijk zijn, genieten, levenslust, veerkracht, gevoel van controle:

a) Krijgt u hierbij hulp?
- ☐ Ja *(ga naar vraag b)*
- ☐ Nee *(ga naar vraag c)*

b) Sluit de hulp die u krijgt goed aan bij wat u nodig heeft?
- ☐ Ja
- ☐ Nee

c) Wilt u hierbij hulp?
- ☐ Ja
- ☐ Nee

D. Zorg en ondersteuning

29. Kunt u aangeven hoe vaak u in **de afgelopen 3 maanden** van de volgende typen personen zorg of ondersteuning heeft gekregen in verband met uw COPD of hartfalen?

	Dagelijks	Wekelijks	Maandelijks	Niet
Directe familie (ouders, echtgenoot, partner, kinderen)	☐	☐	☐	☐
Overige familie (broers, zussen, ooms, tantes, etc.)	☐	☐	☐	☐
Vrienden/buren	☐	☐	☐	☐
Collega's op het werk	☐	☐	☐	☐
Leden van een gemeenschap, club, of vereniging (geloof, sport, hobby, etc.)	☐	☐	☐	☐
Vrijwilligers	☐	☐	☐	☐
Wijkverpleegkundige/thuiszorg	☐	☐	☐	☐
Fysiotherapeut/ergotherapeut	☐	☐	☐	☐
Anders, namelijk:	☐	☐	☐	☐
....	☐	☐	☐	☐
....	☐	☐	☐	☐

30. Kunt u aangeven hoe vaak u in **het afgelopen jaar** van de volgende typen personen zorg of ondersteuning heeft gekregen in verband met uw COPD of hartfalen?

	Maande-lijks	3-4 keer per jaar	2 keer per jaar	1 keer per jaar	Niet
Huisarts of praktijkondersteuner	☐	☐	☐	☐	☐
Arts (cardioloog, longarts) of verpleegkundig specialist in het ziekenhuis	☐	☐	☐	☐	☐
Tandarts	☐	☐	☐	☐	☐
Apotheker	☐	☐	☐	☐	☐
Maatschappelijk werker	☐	☐	☐	☐	☐
Diëtist	☐	☐	☐	☐	☐
Psycholoog/psychiater/psychotherapeut	☐	☐	☐	☐	☐
Alternatieve zorgverleners (homeopaat, acupuncturist, etc.)	☐	☐	☐	☐	☐
Anders, namelijk:	☐	☐	☐	☐	☐
....	☐	☐	☐	☐	☐
....	☐	☐	☐	☐	☐

Draft

ID

31. Bij welke zorgverlener heeft u **de afgelopen drie maanden** de meeste afspraken gehad?

- ☐ Arts of praktijkondersteuner in huisartsenpraktijk
- ☐ Arts (bijvoorbeeld cardioloog of longarts) of verpleegkundig specialist in ziekenhuis
- ☐ Ik heb geen afspraken met zorgverlener(s) gehad
- ☐ Anders, namelijk: ....

32. Als u terugdenkt aan de afspraken met deze zorgverlener:

	**Helemaal geen moeite** 0	1	2	3	4	5	6	7	8	**Alle mogelijke moeite** 9
a) Hoeveel moeite is er gedaan om te luisteren naar de dingen die voor u belangrijk zijn als het gaat om uw **gezondheidssituatie**?	☐	☐	☐	☐	☐	☐	☐	☐	☐	☐
b) Hoeveel moeite is er gedaan om te luisteren naar de dingen die voor u belangrijk zijn in uw **persoonlijke leefsituatie** (uw thuissituatie, werk of woonomstandigheden)?	☐	☐	☐	☐	☐	☐	☐	☐	☐	☐
c) Hoeveel moeite is er gedaan om u te helpen uw gezondheidsituatie te begrijpen?	☐	☐	☐	☐	☐	☐	☐	☐	☐	☐
d) Hoeveel moeite is gedaan om de dingen die voor u belangrijk zijn mee te nemen bij het kiezen van een volgende stap in de behandeling van uw COPD of hartfalen?	☐	☐	☐	☐	☐	☐	☐	☐	☐	☐

33. Bekijkt u alstublieft de volgende vijf afbeeldingen. Welke afbeelding sluit het beste aan bij hoe u wilt dat beslissingen bij de behandeling van uw COPD of hartfalen worden genomen?

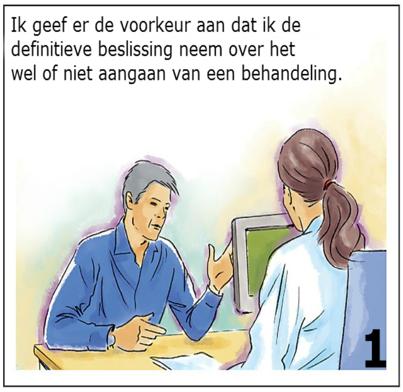

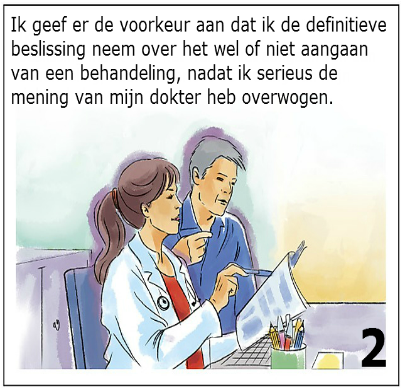

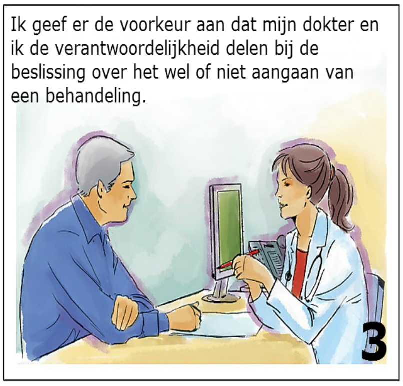

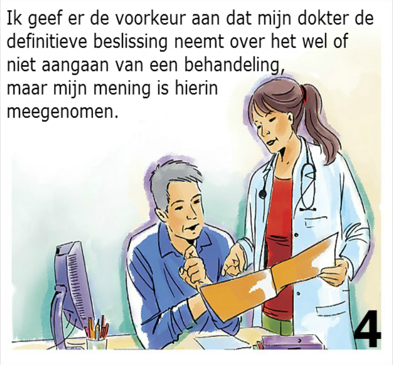

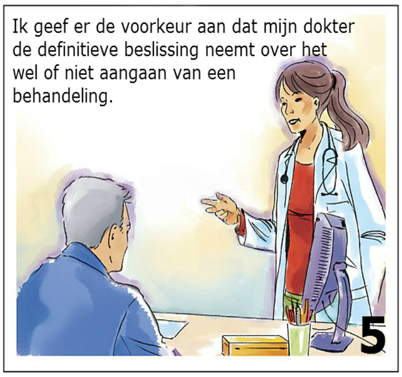

☐ Afbeelding 1 ☐ Afbeelding 2 ☐ Afbeelding 3 ☐ Afbeelding 4 ☐ Afbeelding 5

E. Sociale ondersteuning en dienstverlening

34. Van welke typen ondersteuning heeft u in **het verleden** gebruik gemaakt?

a) Mobiliteitsondersteuning (scootmobiel, rolstoel, rollator, autoaanpassing, etc.)

☐ Ja
☐ Nee

b) Woonaanpassingen (traplift, douchestoel, etc.)

☐ Ja
☐ Nee

c) Juridische ondersteuning (pro deo advocaat, juridisch loket)

☐ Ja
☐ Nee

d) Ondersteuning bij huisvesting en basisvoorzieningen (gas, water, elektriciteit)

☐ Ja
☐ Nee

e) Ondersteuning bij geldzaken (betalen van rekeningen, aanvragen van een uitkering, schuldsanering)

☐ Ja
☐ Nee

35. Kunt u aangeven of, en hoe vaak, u in **de afgelopen 3 maanden** de onderstaande type(n) zorg en ondersteuning heeft gekregen?

	Dagelijks	Wekelijks	Maandelijks	Niet
Ondersteuning bij vervoer (Avan, zorg/regiotaxi, etc.)	☐	☐	☐	☐
Huishoudelijke ondersteuning (schoonmaken, boodschappen, koken, strijken, de was doen, etc.)	☐	☐	☐	☐
Persoonlijke verzorging (wassen, aankleden, toiletgang, etc.)	☐	☐	☐	☐
Ondersteuning bij opleiding, werk, re-integratie	☐	☐	☐	☐
Ondersteuning bij dagactiviteiten voor volwassenen (kunst, handarbeid, muziek, spellen, etc.)	☐	☐	☐	☐
Anders, namelijk:	☐	☐	☐	☐
....	☐	☐	☐	☐
....	☐	☐	☐	☐

36. Als u denkt aan reizen in de stad of het dorp voor zaken als boodschappen doen, afspraken met een arts of het bezoeken van vrienden of familie:

a) Krijgt u hierbij hulp?

☐ Ja *(ga naar vraag b)*
☐ Nee *(ga naar vraag c)*

b) Sluit de hulp die u krijgt goed aan bij wat u nodig heeft?

☐ Ja
☐ Nee

c) Wilt u hierbij hulp?

☐ Ja
☐ Nee

F. Sociale relaties en leefomstandigheden

37. Uw relatie met familie en vrienden kunnen invloed hebben op uw gezondheid en kwaliteit van leven. De volgende uitspraken gaan over uw relatie met familie en vrienden.

	Nooit of zelden	Af en toe	Meestal
a) Mijn familie en vrienden begrijpen mij.	☐	☐	☐
b) Ik voel mij nuttig voor mijn familie en vrienden.	☐	☐	☐
c) Ik ben op de hoogte van hoe het met mijn familie en vrienden gaat.	☐	☐	☐
d) Ik heb het gevoel dat er naar mij geluisterd wordt als ik met familieleden of vrienden spreek.	☐	☐	☐
e) Ik heb een duidelijke plek binnen mijn familie- en vriendenkring.	☐	☐	☐
f) Ik kan mijn meest gevoelige zorgen en problemen delen met minstens één familielid of vriend(in).	☐	☐	☐

38. Als u denkt aan het behouden en/of het verbeteren van uw **sociale leven,** zoals erbij horen, met anderen samen leuke dingen doen, zinvolle dingen doen, iets kunnen betekenen voor anderen:

a) Krijgt u hierbij hulp?

☐ Ja *(ga naar vraag b)*

☐ Nee *(ga naar vraag c)*

b) Sluit de hulp die u krijgt goed aan bij wat u nodig heeft?

☐ Ja

☐ Nee

c) Wilt u hierbij hulp?

☐ Ja

☐ Nee

39. Naast uw relatie met familie en vrienden kan de omgeving/buurt waar u woont invloed hebben op uw gezondheid en kwaliteit van leven. De volgende uitspraken gaan over de omgeving/buurt waar u woont.

	Zeer eens	Eens	Oneens	Zeer oneens
a) Dit is een hechte buurt.	☐	☐	☐	☐
b) Mensen hier zijn bereid om buren te helpen.	☐	☐	☐	☐
c) Mensen in deze buurt delen niet dezelfde waarden.	☐	☐	☐	☐
d) Mensen uit deze buurt zijn te vertrouwen.	☐	☐	☐	☐
e) Het is veilig op straat, bij mij in de buurt.	☐	☐	☐	☐

40. Als u denkt aan het behouden en/of het verbeteren van uw **persoonlijke veiligheid,** zoals bescherming tegen geweld, bedreigingen, lastig worden gevallen en de kans op vallen:

a) Krijgt u hierbij hulp?

☐ Ja *(ga naar vraag b)*

☐ Nee *(ga naar vraag c)*

b) Sluit de hulp die u krijgt goed aan bij wat u nodig heeft?

☐ Ja

☐ Nee

c) Wilt u hierbij hulp?

☐ Ja

☐ Nee

41. Uw woonsituatie kan invloed hebben op uw gezondheid en kwaliteit van leven. Wat vindt u van:

	Prima	Goed	Redelijk	Niet goed/ niet slecht	Matig	Slecht	Vreselijk
a) De omstandigheden waarin u leeft (ruimte, comfort, hygiëne, etc.)?	☐	☐	☐	☐	☐	☐	☐
b) De privacy die u hier heeft?	☐	☐	☐	☐	☐	☐	☐
c) Het vooruitzicht voor langere tijd om te blijven waar u nu woont?	☐	☐	☐	☐	☐	☐	☐

42. Als u denkt aan het behouden en/of het verbeteren van uw **woonsituatie,** zoals huisvesting en woonvoorzieningen:

a) Krijgt u hierbij hulp?

☐ Ja *(ga naar vraag b)*

☐ Nee *(ga naar vraag c)*

b) Sluit de hulp die u krijgt goed aan bij wat u nodig heeft?

☐ Ja

☐ Nee

c) Wilt u hierbij hulp?

☐ Ja

☐ Nee

G. Financiële situatie

43. Had u **het afgelopen jaar** elke maand voldoende geld om uit te geven aan de volgende zaken?

	Ja	Nee
a) Voeding	☐	☐
b) Kleding	☐	☐
c) Woning/onderkomen	☐	☐
d) Medische zorg	☐	☐
e) Reizen in de stad of het dorp voor zaken als boodschappen doen, afspraken met een arts of het bezoeken van vrienden of familie	☐	☐
f) Sociale activiteiten als het uit eten gaan in een restaurant of naar de bioscoop gaan	☐	☐

44. Heeft u in **het afgelopen jaar** financiële ondersteuning gekregen, naast uw eigen inkomen of pensioen?

- ☐ Ja
- ☐ Nee *(sla de volgende vraag over)*

45. Uit welke bronnen kwamen in **het afgelopen jaar** uw inkomsten?

	**Ja**	**Nee**	**Weet ik niet**
a) Geld van familie en/of vrienden	☐	☐	☐
b) Uitkering(en) van de overheid: bijstandsuitkering; daklozenuitkering; uitkering wegen ziekte of arbeidsongeschiktheid (WAO, AAW, ziektewet, WIA, Wajong), werkeloosheid (WW, IOAW, IOW, IOAZ)	☐	☐	☐
c) Sociale werkvoorziening loon	☐	☐	☐
d) Huurtoeslag	☐	☐	☐
e) Bijdragen en alimentatie voor kinderen	☐	☐	☐
f) Voedsel van voedselbank en/of van andere personen of instanties	☐	☐	☐
g) Zorgtoeslag (collectieve aanvullende ziektekostenverzekering: CAZ)	☐	☐	☐
h) Anders, namelijk	☐	☐	☐

46. Als u denkt aan het regelen van uw **geldzaken -** zoals het betalen van rekeningen, aanvragen van een uitkering, terugbetalen van een schuld of lening:

a) Krijgt u hierbij hulp?

- ☐ Ja *(ga naar vraag b)*
- ☐ Nee *(ga naar vraag c)*

b) Sluit de hulp die u krijgt goed aan bij wat u nodig heeft?

- ☐ Ja
- ☐ Nee

c) Wilt u hierbij hulp?

- ☐ Ja
- ☐ Nee

47. Als u denkt aan toegang tot **etenswaren/maaltijden**:

a) Krijgt u hierbij hulp?

- ☐ Ja *(ga naar vraag b)*
- ☐ Nee *(ga naar vraag c)*

b) Sluit de hulp die u krijgt goed aan bij wat u nodig heeft?

- ☐ Ja
- ☐ Nee

c) Wilt u hierbij hulp?

- ☐ Ja
- ☐ Nee

U heeft de vragenlijst afgerond.

Hartelijk dank voor uw deelname aan dit onderzoek!

Voor dit onderzoek willen wij graag een aantal patiënten met COPD en/of hartfalen interviewen. Met deze interviews willen wij beter begrijpen waarom patiënten ontevreden of juist heel tevreden zijn met de huidige zorg en ondersteuning die zij krijgen. Daarnaast willen wij beter begrijpen wat patiënten vinden van hun rol en de inbreng die zij hebben in de behandeling van hun chronische ziekte.

48. Mogen wij u voor een interview benaderen? Het interview zal ongeveer 30 tot 60 minuten duren. U kunt zelf bepalen waar het interview plaatsvindt (bijvoorbeeld thuis of in het Radboudumc).

☐ Ja   Mijn telefoonnummer/e-mailadres is ………………………………………………………

☐ Nee

Draft

ID
