# Supplementary material for: An international comparison of factors affecting quality of life among patients with congestive heart failure: A cross-sectional study
Source: PLoS One. 2020 Apr 8;15(4):e0231346. doi: 10.1371/journal.pone.0231346 (PMC7141662; doi:10.1371/journal.pone.0231346)
Supplement: S2 File — (DOC) [file pone.0231346.s002.doc]

**Rhelaunch Pilot Survey**

Hello. My name is . I'm working on a study to better understand the quality of life and daily experiences of patients with COPD and/or heart failure. We’d like to better understand how healthcare and social services contribute to patients' health and quality of life and would like to ask you some questions about your health, healthcare, social support, and social services you receive.

We’ll combine your responses with responses from other patients here at HCMC and at a similar hospital in the Netherlands to understand differences between the two countries and how we can improve healthcare for patients. All of your answers will be confidential and never associated with your name. Your participation in this survey will not affect any of the healthcare or social services you receive.

The survey will take about 45 minutes. Some people need more time, some people need less time. Participation is completely voluntary and you do not have to answer any question you do not want to. Would you be willing to answer some questions about your health, healthcare, and social situation?

[if yes,]

Great. Don't think about the questions too long; there are no right or wrong answers. Please choose the answer that matches best with your situation.

If you have any questions or comments about this survey, you can call Dr. Bart at 612-873-2875. [if no,]

That&rsquo;s ok, we&rsquo;ll see you at your next appointment.

If you have any questions or comments about this survey, you can call Dr. Bart at 612-873-2875.

site ID

# SECTION 1Physical healthFirst we'll start with some questions about your physical health, lifestyle, and daily functioning.

In general how would you rate your physical health?

excellent very good good fair poor did not answer

# SECTION 1, continuedPlease tell us how much trouble you've had doing these daily activities during the past 4 weeks. The options are not at all, very little, a little, moderate, quite a bit, very much, or extremely.

walking upstairs running a short distance cycling

lifting a heavy object going to the toilet

taking a bath or shower, and/or getting dressed

not at all very little a little moderat

ely

quite a bit

very much

extremel y

do not do this activity

did not answer

cleaning the house looking after family shopping

# SECTION 2

**Mental health**

**Ok, now I'm going to ask you a few questions about your mental health.**

In general, how would you rate your mental health, including your mood and ability to think?

excellent very good good fair poor did not answer

# SECTION 2, continuedWe are interested in how you deal with stress and setbacks in life. Please consider how well the following statements describe your behavior and actions. Do you strongly agree, agree, neither agree nor disagree, disagree, or strongly disagree.

I feel that I mean something to someone else

strongly agree agree neither agree

nor disagree

disagree strongly disagree

did not answer

My life has purpose.

When things go well, I feel satisfied.

I am able to deal with the problems that come my way.

I decide how I control my life. I am determined to go on.

My chronic illness does not define my identity.

I think of myself as a person worth something.

I turn negative thoughts into positive ones.

I can see how my life has made me who I am today.

I can deal with my vulnerabilities. I am not afraid to rely on myself.

# SECTION 2, continuedNow I'm going to ask you a few sensitive questions about your experiences with tobacco, alcohol, and other drugs. Your answers won't be shared with anyone outside this research project.

In the past year, how often have you used any tobacco products, for example: cigarettes,

e-cigarettes, cigars, pipe, or smokeless tobacco?

In the past year, how often have you had 5 or more drinks containing alcohol in one day?

daily or almost daily

weekly monthly less than monthly

never did not answer

In the past year, how often have you used any drugs including: marijuana, cocaine or crack, heroin, methamphetamine (crystal meth), hallucinogens, or ecstasy/MDMA?

In the past year, how often have you used any prescription medications just for the feeling, more than prescribed, or that were not prescribed to you?

# SECTION 3

**Use of care and support**

**Now I'm going to read you a list of people who may have provided care or support in the last three months. You may have received care or support from several groups on the list. Please estimate how often you received care or support from each of the following; daily, weekly, monthly, or not at all.**

immediate family (parent, sibling, spouse, partner, children)

daily weekly monthly never did not answer

other family members (aunt, uncle, cousins, etc.)

friends/neighbors work colleague

community organizations (faith-based, internet support groups, community centers, sports groups, hobby groups, food pantries, etc.)

volunteers

primary care clinic (e.g., general practitioner, nurse practitioner, physician assistant)

specialty care clinic (e.g., cardiology or pulmonary: specialist physician, nurse practitioner, physician assistant)

home visit nurse dentist

social worker pharmacist

psychologist/psychiatrist/ psychotherapist

physical therapist/occupational therapist

dietitian

complementary/alternative medicine (e.g., acupuncturist, chiropractor)

other, please specify

Please specify who you received care or support from.

# SECTION 3, continued

**Now I'm going to ask you a few questions about your experiences with health care providers.**

I'm going to read you a list of care providers, which primary care (e.g., general practitioner, nurse provider have you had the most appointments with in practitioner, physician assistant)

the last 3 months? specialist physician, nurse practitioner,

physician assistant (e.g. cardiology, pulmonology) community or home care nurse

physical therapist other, please specify

have not used any of these services did not answer

Please specify the provider you had the most appointments with in the last 3 months

# Please think about the appointments with this care provider as I read you a few questions. Answer the questions on a scale of 0 to 9, with 0 being no effort was made to 9, every effort was made.

How much effort was made to listen to the things that matter most to you about your heath?

0 no effort was made

1 2 3 4 5 6 7 8 9

every effort was made

did not answe r

How much effort was made to listen to the things that matter most to you about your personal life situation (e.g. household, living conditions, work, family, friends, finances, etc.)?

How much effort was made to help you understand your health issues?

How much effort was made to include what matters most to you in choosing what to do next in the treatment of your COPD/heart failure?

# SECTION 3, continued

Okay, now I'm going to show you some images. Which A

image most accurately reflects your preferences B

regarding decision making about treatment of your C

COPD/heart failure? D

E

did not answer

[Inline Image: "rhelaunch survey pic Q33.jpg"]

# SECTION 4

**Social support**

**Now I'm going to ask you some questions about social support.**

Have you received support for mobility aids (mobility Yes

scooter, wheelchair, etc)? No

did not answer

Have you received support with fitting equipment and Yes

adaptations to the home (stair lifts, bath seats, No

etc.)? did not answer

Have you received legal help or support? Yes No

did not answer

Have you received help with utilities (water, Yes

electricity, gas, heat)? No

did not answer

Have you received advice and help with money from a Yes

professional or agency (paying bills, debt or loan No

repayment, applying for public benefits)? did not answer

# Now I'm going to ask you some questions about social support you might have received over the last three months.

In the past three months, how often did you get support with free or low-cost transportation services (bus, taxi,etc.)?

daily weekly monthly never did not answer

In the past three months, how often did you get support with home/household aid (cleaning, shopping, food prep, laundry, etc.)?

In the past three months, how often did you get support with personal care aid (getting in and out of bed, bathing, using the toilet, etc.)?

In the past three months, how often did you get support with employment, work, education?

In the past three months, how often did you get support with adult daycare services (art, music, game activities, etc)?

In the past three months, how often did you get other social support?

Please specify any other social support you received.

# SECTION 5

**Social relationships and living situation**

**Okay, great. Now I'm going to ask about your relationships and living situation. The following statements are about your relationships with friends and family; do you feel this way hardly ever, some of the time, or most of the time?**

My family and friends (people who are important to me) seem to understand me.

hardly ever some of the time most of the time did not answer

I feel useful to my family and friends (people who are important to me).

I know what is going on with my family and friends.

When I talk with my family and friends, I feel I am being listened to.

I feel I have a definite role (place)

in my family and among my friends.

I can talk about my deepest

problems with at least some of my family and friends.

# SECTION 5, continuedIn addition to your social relationships, the neighborhood you live in also has an impact on your health, social support, and quality of life. When thinking about the place you live, do you strongly agree, agree, disagree, or strongly disagree?

This is a close-knit neighborhood.

People around here are willing to help their neighbors.

strongly agree agree disagree strongly disagree did not answer

People in this neighborhood do not share the same values.

People in this neighborhood can be trusted.

It is safe on the streets of my neighborhood.

# SECTION 5, continued

**When thinking about the place you live, how do you feel about the following areas:**

The living arrangements where you live (comfort, space, etc.)

delighted happy mostly

satisfied

mixed mostly dissatisfie

d

unhappy terrible did not

answer

The privacy you have there

The prospect of staying where you currently live for a longer period of time

# SECTION 6

**Quality of life and socioeconomic status**

**For the next 2 questions please imagine a ladder with steps numbered from 0 at the bottom to 10 at top.**

| The top of the ladder (10) represents the best | 10 |
| --- | --- |
| possible life for you and the bottom of the ladder | 9 |
| (0) represents the worst possible life for you. On | 8 |
| which step of the ladder would you say you personally | 7 |
| stand at this time? | 6 |
|  | 5 |
|  | 4 |
|  | 3 |
|  | 2 |
|  | 1 |
|  | 0 |
|  | did not answer |

| [Inline Image: "rhelaunch survey pic ladder Qs.jpg"]  Now imagine the top of the ladder (10) represents the | 10 |
| --- | --- |
| best possible financial situation for you, and the | 9 |
| bottom of the ladder (0) represents the worst | 8 |
| possible financial situation for you. Please indicate | 7 |
| where on the ladder you stand right now. | 6 |
|  | 5 |
|  | 4 |
|  | 3 |
|  | 2 |
|  | 1 |
|  | 0 |
|  | did not answer |

[Inline Image: "rhelaunch survey pic ladder Qs.jpg"]

# SECTION 7

**Demographics**

**[need transition language]**

What is your zip code?

What is your marital status? single (never married or never in a same-sex civil partnership)

married (first marriage) re-married

in a registered same-sex civil partnership separated

divorced widowed

did not answer

What is your race/ethnicity? Please select all that White

apply. Black or African American

Hispanic/Latino or Spanish origin American Indian or Alaska Native Asian

Native Hawaiian or other Pacific Islander did not answer

Are you covered by health insurance or some other yes

kind of health care plan? no

don't know/unsure did not answer

What type of health insurance coverage do you have? insurance through your job

private insurance (MNsure, Obamacare, Affordable Care Act, health insurance exchange, etc.) Medicaid

Medicare don't know

did not answer

Is the place where you live: owned by you or someone else in the household rented for money

occupied without payment of money or rent homeless

other

did not answer

Who else lives with you? More than one answer is no one besides myself

possible. significant other/partner/spouse

children parent(s)

I live at the home of one of my children other

did not answer

What is your highest level of education? less than high school

high school diploma or GED some college (no degree) associates degree bachelors degree

masters degree doctorate

professional (MD, JD, DDS, etc.) none of the above

did not answer

Which of the following best describes your current working full time

main daily activities and/or responsibilities? working part time

partial or full incapacity for work unemployed or laid-off (looking for work) keeping house or raising children full time retired

volunteering other

did not answer

# SECTION 8Finances

In the past 12 months, did you receive any financial Yes

support beyond your own earned income or No

retirement/pension? did not answer

# In the past 12 months, from which of the following sources did you receive income or financial support?

Money from family and/or friends

Financial support from the government: Social Security Disability Income (SSDI), Supplemental Security Income (SSI), armed services connected disability payments, other social welfare benefits (general welfare, or Temporary Assistance for Needy Families (TANF))

Yes No did not answer

Vocational program (Workforce Investment Act of 1998 (WIA), vocational rehabilitation, sheltered workshop)

Rent supplements (HUD, Section 8 vouchers, living programs receiving public assistance support)

Alimony or child support Food stamps (SNAP) Other, please specify

Please specify where you receive income or financial support

# SECTION 8, continuedDuring the past year, did you generally have enough money each month to cover:

food clothing housing medical care

traveling around the city for things like shopping, medical appointments, or visiting friends and relatives

social activities like movies or eating in restaurants

yes no did not answer

# SECTION 9

**Needs assessment**

**This is the last set of questions. I'm going to read you a list of things that people sometimes need help with.**

Do you get help with maintaining or improving your Yes

physical functioning - things like your energy level No

or symptoms such as pain and shortness of breath, did not answer etc.

Does the help you get meet your needs? Yes No

did not answer

Would you like help with maintaining or improving Yes

your physical functioning - things like your energy No

level or symptoms such as pain and shortness of did not answer breath, etc.

Do you get help with maintaining or improving your Yes

daily activities - things like personal care, doing No

work or school activities, shopping, hobbies, house did not answer cleaning, etc.?

Does the help you get meet your needs? Yes No

did not answer

Would you like help with maintaining or improving Yes

your daily activities - things like personal care, No

doing work or school activities, shopping, hobbies, did not answer house cleaning, etc.?

Do you get help with maintaining or improving your Yes

mental health - things like being happy, resilient, No

in control, lust for life? did not answer

Does the help you get meet your needs? Yes No

did not answer

Would you like help with maintaining or improving Yes

your mental health - things like being happy, No

resilient, in control, lust for life? did not answer

Do you get help with maintaining or improving your Yes

social life - things like being amongst others, No

participating in society, being meaningful to others, did not answer etc.?

Does the help you get meet your needs? Yes No

did not answer

Would you like help with maintaining or improving Yes

your social life - things like being amongst others, No

participating in society, being meaningful to others, did not answer etc.?

Do you get help with maintaining and/or improving Yes

your personal safety - things like protection against No

violence, harassment, assaults, nuisance, falling? did not answer

Does the help you get meet your needs? Yes No

did not answer

Would you like help with maintaining and/or improving Yes

your personal safety - things like protection against No

violence, harassment, assaults, nuisance, falling? did not answer

Do you get help with housing and utilities (heat, Yes

electricity, water, gas)? No

did not answer

Does the help you get meet your needs? Yes No

did not answer

Would you like help with housing and utilities (heat, Yes

electricity, water, gas)? No

did not answer

Do you get help with transportation? Yes No

did not answer

Does the help you get meet your needs? Yes No

did not answer

Would you like help with transportation? Yes No

did not answer

Do you get help with access to food? Yes No

did not answer

Does the help you get meet your needs? Yes No

did not answer

Would you like help with access to food? Yes No

did not answer

medical supplies? No

did not answer

Does the help you get meet your needs? Yes No

did not answer

Would you like help with medical care, medicine, or Yes

other medical supplies? No

did not answer

Do you get help with applying for public benefits Yes

(WIC, SSI, SNAP, etc.)? No

did not answer

Does the help you get meet your needs? Yes No

did not answer

Would you like help with applying for public benefits Yes

(WIC, SSI, SNAP, etc.)? No

did not answer

Would you like to speak with someone about getting Yes

help for any items we just discussed? No

did not answer

Thank you for completing this survey. We'd like to Yes

ask some additional questions from several volunteers No in the near future to get a better understanding of

the care needs and preferences of patients with chronic diseases, and the reasons why needs are met

or unmet by services. The additional interview will take between 30 and 60 minutes and take place as a location you prefer, like the clinic or your home.

Could we contact you in the future for an interview?
