# Supplementary material for: An international comparison of factors affecting quality of life among patients with congestive heart failure: A cross-sectional study
Source: PLoS One. 2020 Apr 8;15(4):e0231346. doi: 10.1371/journal.pone.0231346 (PMC7141662; doi:10.1371/journal.pone.0231346)
Supplement: S1 Fig — (PDF) [file pone.0231346.s004.pdf]

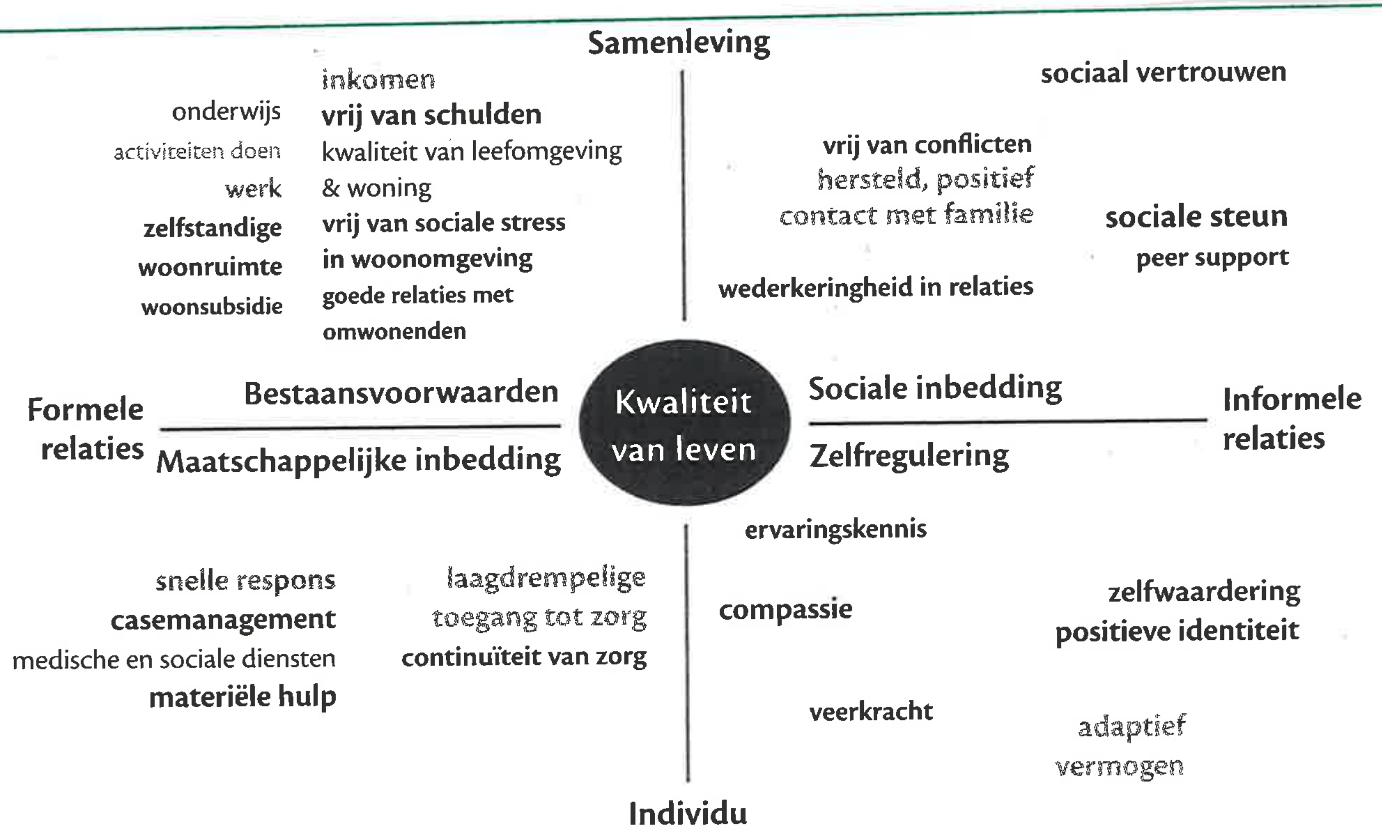

Figuur 6.4
Versterkers van participatie en zelfregie, geordend naar model van sociale kwaliteit (©Wolf)
