# Supplementary material for: An international comparison of factors affecting quality of life among patients with congestive heart failure: A cross-sectional study
Source: PLoS One. 2020 Apr 8;15(4):e0231346. doi: 10.1371/journal.pone.0231346 (PMC7141662; doi:10.1371/journal.pone.0231346)
Supplement: Attachment — Submitted filename: Response to Reviewers_PONE-D-19-33707_R2_FINAL.docx [file pone.0231346.s006.docx]

| Yale SCHOOL OF MEDICINE | **Department of Internal Medicine** |
| --- | --- |
|  | **Section of General Internal Medicine** |
|  | PO Box 208025 |
|  | New Haven, CT 06520-8025 |
| March 19, 2020 | T: 203-785-5564 |
|  | F: 203-737-3306 |
| Hans-Peter Brunner-La Rocca, M.D. | *Courier*: |
| Academic Editor, *PLOS ONE* | Edward S. Memorial Harkness Hall A (ESH) |
| Maastricht University Medical Center | Room 406A |
| Division of Structural Heart Diseases | 367 Cedar Street |
| Maastricht, NL | New Haven, CT 06150 |

Dear Dr. Brunner-La Rocca,

Thank you again for your consideration of our manuscript titled, “An international comparison of factors affecting quality of life among patients with congestive heart failure: A cross-sectional study” (PONE-D-19-33707), for publication in *PLOS ONE*. Below we have responded directly to the remaining editor’s and reviewer’s comments and we have specified where we have made changes to the text based on these comments. All line numbers refer to those in the file labeled ‘Revised Manuscript with Track Changes.’

**Editor**: *I would like to ask the authors to include the lack of data mentioned by reviewer #2 as a limitation to the study.*

**Reviewer 2:** *The authors have appropriately responded to the reviewers' comments. I was thinking that a better characterization of the patients in terms of measures of cardiac function, functional capacity, comorbidities and medication would have strengthened the paper. Obviously, this data is not available, and the authors argue that the findings can be properly interpreted even in absence of this information. I think that it is up to the editors to decide whether the paper is acceptable despite this Limitation.*

Response: We have added the following text to the limitations paragraph to directly address the constraints of our comparable data (lines 420-424): “participants in the US were more likely to report having at least one other medical condition and being prescribed at least six medications. As such, it is likely US participants were sicker than NL participants, but we did not have comparable data on detailed clinical information, such as left ventricular ejection fraction, brain natriuretic peptide, device therapy, and specific comorbid medical conditions to further characterize these differences.”

Again, we thank you for the opportunity to revise our manuscript and we hope you will find this version suitable for publication in *PLOS ONE*.

Sincerely,

Brita Roy, MD, MPH, MHS

Assistant Professor of Medicine and Epidemiology
